# Characterizing the circadian connectome of *Ocimum tenuiflorum* using an integrated network theoretic framework

**DOI:** 10.1038/s41598-023-40212-7

**Published:** 2023-08-11

**Authors:** Vikram Singh, Vikram Singh

**Affiliations:** Centre for Computational Biology and Bioinformatics, Central University of Himahcal Pradesh, Dharamshala, Himahcal Pradesh 176206 India

**Keywords:** Computational biology and bioinformatics, Network topology, Proteome informatics

## Abstract

Across the three domains of life, circadian clock is known to regulate vital physiological processes, like, growth, development, defence etc. by anticipating environmental cues. In this work, we report an integrated network theoretic methodology comprising of random walk with restart and graphlet degree vectors to characterize genome wide core circadian clock and clock associated raw candidate proteins in a plant for which protein interaction information is available. As a case study, we have implemented this framework in *Ocimum tenuiflorum* (Tulsi); one of the most valuable medicinal plants that has been utilized since ancient times in the management of a large number of diseases. For that, 24 core clock (CC) proteins were mined in 56 template plant genomes to build their hidden Markov models (HMMs). These HMMs were then used to identify 24 core clock proteins in *O. tenuiflorum*. The local topology of the interologous Tulsi protein interaction network was explored to predict the CC associated raw candidate proteins. Statistical and biological significance of the raw candidates was determined using permutation and enrichment tests. A total of 66 putative CC associated proteins were identified and their functional annotation was performed.

## Introduction

Earth’s rotation around its axis and the sun generates repetitive changes in the environmental conditions, like, the dark-light cycle, the rhythmicity of light intensity, temperature, seasons etc. Guided by these environmental changes, living organisms possess biological rhythms called circadian rhythms, which are endogenously generated, self-sustained, and temperature compensated and have a period approximately equal to 24 h in free-running conditions^1–4^. Circadian rhythms directly influence the physiological processes of all living beings, including plants, that are helpful in the diurnal modulation of biological events^5^. The involvement of the circadian clock in regulating vital plant processes, like, nutrient biogenesis, root growth, photosynthesis, hormonal signalling, sugar metabolism, flowering time, and plant immunity, has been documented in many studies^6–10^. These biological rhythms are entrained with their external environment by an internal timekeeper called a circadian clock that keeps track of environmental fluctuations and allows temporal separation of internal physical events accordingly^11^. The circadian clock is crucial for optimizing a plant’s adaptive responses, in different physiological processes, including growth, development and defence, to varying environmental conditions and improving the overall fitness of the plant^8,12^.

Circadian rhythms in most life forms are governed by a set of 20–24 genes called core circadian clock (CC) genes which regulate vital life-sustaining processes through another set of genes called core circadian clock associated (CCa). Thus the complete circadian pathway comprises the core clock genes and genes involved in downstream processes^13^. It has been shown that circadian oscillators are ubiquitous among all three domains of life with minimal elemental (at gene level) conservation across them, however, their architectures and mechanisms are remarkably convergent across domains^14–16^. Recent advancements in high-throughput next generation sequencing platforms have revolutionised the practice of modern biology by generating a massive volume of molecular sequence data^17^ that can be leveraged to characterize core circadian clock (CC) and clock associated genes at systems scale^18^. The dynamics of core circadian clock genes under different stress conditions have recently been characterised in *Camelia sinensis*^19^, however, comprehensive network theoretic methodology to characterise various core circadian clock associated genes is still lacking. Knowing the identities of genes involved in important downstream pathways will allow us to optimally utilize the cellular circuitry for crop improvement and increase the production of essential oils. Since the number of genes involved in downstream pathways is very large, functioning independently in several distinct sub-modules, studying them together is highly difficult through a reductionist approach. Network science provides a way to explore these interactions at a systems scale and draw conclusions from them.

Topologically similar proteins in protein interaction networks (PINs) have been shown to perform similar functions^20^. Several approaches have shown that the architectural properties of proteins in an interactome complement their structural and sequence properties and could be successfully utilized for inferring the functional annotations of interacting proteins^21–23^. The present study reports a novel integrated network framework that entirely relies on network topology to characterize rhythmic genes in plants. Various statistical approaches, including LS^24^, ARSER^25^, JTK_CYCLE^26^, RAIN^27^, MetaCycle^28^, BIO_CYCLE^29^ for characterizing oscillatory genes along with their estimated rhythmic parameters *i.e.* phase, amplitude and waveform have been proposed^30^. All these approaches utilize temporal transcriptomic data to characterize circadian regulated genes and estimate their rhythmic parameters. Dealing with transcriptomic data is time and labour-intensive and demands extensive computational capacity that is only sometimes feasible. On the other hand, our approach is highly time and memory efficient and requires minimum resources.

*Ocimum tenuiflorum* (Tulsi) is a member of family Lamiaceae, an aromatic herb that is widely recognized for array of health benefits it offers^31^. We find references of the holy basil in Ayurveda, an ancient medicinal system, for its usage in treating variety of diseases and disorders either independently or as a formulation in combination with several other herbs^32^. The plant is reared for its oils and every part of this plant produces a number of secondary metabolites which possess significant pharmaceutical properties^33^. Recently, genome^34,35^, tanscriptome^36^ and proteome^37^ wide studies have reported a draft catalogue of genes, transcripts and proteins present in Tulsi along with important mechanistic insights about the secondary metabolite biosynthetic pathways^38,39^. However, an exhaustive systems scale study presenting core circadian clock and clock associated genes along with complex interplay among them at molecular level is still lacking in *O. tenuiflorum*. This motivated us to exploit the topology of the Tulsi protein interaction network^37^ to identify circadian regulated genes. In this study, we propose a systems scale approach to mine and characterize core circadian clock proteins giving rise to these rhythms in *O. tenuiflorum*. We leveraged random walk with restart (RWR) and graphlet degree vector (GDV) methods to explore the local topology of protein interaction network of Tulsi (TulsiPIN) and identified raw candidate proteins associated with the core oscillator. Furthermore, statistical and biological significance of the raw candidates was determined and their functional annotations were determined.

## Materials and methods

### Data collection

From the literature, 24 core circadian clock (CC) proteins of *Arabidopsis thaliana*^10,13^ were identified, and their sequences were retrieved from the TAIR database^40^. Proteome data of 56 template plants, for which genome information is available, was downloaded from the UniProt database https://www.uniprot.org/. All the CC proteins were then queried against each template proteome using Blast-P to identify homologous proteins. Top hits were selected for each CC sequence having E value $\le 10^{-5}$, with at least $40 \%$ sequence identity and $50 \%$ query coverage. CC sequence specific multiple sequence alignments were then obtained using Clustal Omega^41^. HMMER http://hmmer.janelia.org/ was used to construct Hidden Markov models for each CC protein. These HMMs were then used to scan *O. tenuiflorum* proteome obtained from TulsiDB http://caps.ncbs.res.in/Ote/ to identify putative CC proteins in Tulsi. Each identified CC protein was subjected to InerProScan v5.48-83 https://www.ebi.ac.uk/interpro/ to identify their respective domains^19^. Protein-protein interaction network of *O. tenuiflorum* called TulsiPIN, developed in our previous work^37^, was considered for network scale studies. TulsiPIN is an exhaustively constructed network consisting of 13,604 nodes and 327,374 edges with scale free and small world properties.

### Mining core circadian clock associated (CCa) proteins using local topological measures

Two local network topology based algorithms, namely, random walk with restart (RWR) and graphlet degree vector (GDV) were leveraged to identify core circadian clock associated proteins.

#### Random walk with restart (RWR) based mining

RWR is a ranking algorithm that has been successfully applied to estimate the likelihood of genes/proteins being associated with a given biological process (X) based on their proximity to already known proteins belonging to X in the input network^42^. The algorithm initiates a specific number of walks from one or more seed nodes (CC proteins) to some random nodes in the network and returns the probability of each protein being a CCa protein. This study applied the RWR algorithm on TulsiPIN using 9 CC proteins (which could be mapped to TulsiPIN) as seed nodes. Initially, a vector $P_0$, containing 13, 604 elements, each corresponding to a node in TulsiPIN, was constructed in which each CC protein was initialized with a number $\frac{1}{9}$ and other proteins with zero. The vector is then updated using the equation $P_{i+1} = (1-r) A^T P_i + rP_0$ for *i*th iteration until $P_i$ is stabilized. Here, *r* represents restart probability that is set as (0.8), and *A* is TulsiPIN’s adjacency matrix every column of that sums up to one. The iterations stop when Manhattan distance between $P_i+1$ and $P_i$ is less than $10^{-6}$ i.e. $||P_{i+1} - P_i||_{L_1} < 10^{-6}$. This yielded a probability vector containing the probability values corresponding to each protein of TulsiPIN, quantifying their likelihood of being CC associated. Accordingly, a threshold value of $10^{-5}$ was adopted and all the proteins having probabilities higher than the selected threshold, called raw CCa proteins, were considered for further study^43^.

#### Graphlets degree vector (GDV) based mining

Small, non-isomorphic, induced sub-graphs of a large network are defined as graphlets^44^. Based on the node position within a graphlet, each sub-graph can be differentiated into symmetrically equivalent sets of nodes (symmetry groups) called automorphism orbits^45^. A total of 15 automorphism orbits are possible for up to 4 node graphlets. Each node of an undirected network can be represented with a vector of length 15, each element of which represents the degree of the corresponding orbit, called graphlet degree vector (GDV). Finally, a vector of 11 non-redundant orbits^46^ containing orbit degrees, representing wiring around that node, was computed for every node in the network. Since current PPI networks have been shown to be largely incomplete and lower degree nodes are more likely to be present in those incomplete parts, we remove proteins with degrees lower than three^22^. Among the 24 CC proteins, only 10 could be mapped to TulsiPIN. Since one had a degree lower than three, so removed from the further study. For each of 9 CC proteins, a pairwise similarity ($S_{GDV}$) was computed with every TulsiPIN protein having a degree more than three that is defined as

$$\begin{aligned} S_{GDV} = 1-\frac{\sum |u_i - v_i |}{\sum |u_i + v_i |} \end{aligned}$$

where *u* and *v* represent GDV of CC proteins and TulsiPIN proteins respectively, while *i* represents the *i*th orbit. All proteins with similarity above $94 \%$ with the protein of interest were considered CCa proteins. Previously a signature similarity threshold within the range 90–95% has been shown to be optimal^22^. So to select the signature similarity threshold, we started with a threshold value of $96\%$ and decreased the threshold value until we obtained at least one TulsiPIN protein similar with each CC protein other than themselves.

### Statistical and functional validation of CCa proteins

As these algorithms exploit the topology of the network to estimate the likelihood, this may output some false positive proteins as CCa proteins *e.g.* hubs will be ranked higher than those having degree lower than the average degree of network^43^. To reduce these false positive proteins and assess the reliability of each prediction, two methods, namely, permutation test and enrichment test were applied.

#### Permutation or randomisation test

To test the statistical significance of CCa proteins identified by RWR algorithm, we sampled 1000 random sets of 9 proteins each from 13,604 TulsiPIN proteins. RWR algorithm was then performed on TulsiPIN using each of these samples as a seed. A *p* value or permutation FDR for every CCa protein predicted was computed based on the following equation $p (g) = \frac{\theta _{RWR}}{1000}$, where $\theta _{RWR}$ is the number of random seeds for which probability of *g*, a CCa protein *p*(*g*), is higher than *p*(*g*) obtained using CC proteins as seed to RWR algorithm. On the other hand, to test the statistical significance of CCa proteins obtained from the GDV algorithm, we first constructed an ensemble of 1000 random networks with the same number of nodes and similar degree sequence^47^ and then computed graphlet degree vectors for each protein of every network. Then pairwise $S_{GDV}$ similarity scores were computed for each CC protein with other proteins in TulsiPIN. Similar to RWR, a permutation FDR for every CCa protein predicted by the GDV algorithm was computed using $p (g) = \frac{\theta _{GDV}}{1000}$, where $\theta$ is the number of random networks for which the similarity score of protein pair between *g*, a CCa gene, and some CC gene is higher than the similarity score obtained for same pair in the real network. Higher *p* value of a gene indicates the gene is less likely to be associated with CC. Thus CCa proteins with *p* value less than 0.05 were selected from both methods, and the selected proteins were called candidate CCa proteins for convenience.

#### Enrichment test

The enrichment test is based on the premise that interacting proteins are more likely to share common functions^37,48^. So, in this section, we used PPI information from TulsiPIN and Gene Ontology (GO) and KEGG Orthology (KO) annotation information of interacting proteins to infer the validity of candidate CC associated proteins. It has been shown earlier that similar proteins may perform similar molecular functions, localize in the same cellular milieu and may be involved in similar biological processes^37,48^. Thus candidate CCa proteins having similar functional annotations as that of CC proteins may be the novel CCa proteins. To quantify the relationship between a protein and an annotation term (GO or KEGG pathways), an ontology enrichment score, the negative log of probability value obtained from the hypergeometric test was computed^49^. Given *N* total TulsiPIN proteins, among which *M* are annotated by one annotation term (*A*), if we randomly draw a set of proteins (*H*(*g*)) containing any protein *g* and its immediate neighbours, then the probability that at least *m* of them are annotated with *A* is given by

$$\begin{aligned} S(g,A) = -log_{10} \left( \sum _{{k=m}}^{n} \frac{\left( {\begin{array}{c}M\\ k\end{array}}\right) \left( {\begin{array}{c}N-M\\ n-k\end{array}}\right) }{\left( {\begin{array}{c}N\\ n\end{array}}\right) } \right) \end{aligned}$$

where *n* is the number of proteins present in set *H*(*g*) and *m* is the number of *H*(*g*) proteins annotated by *A*. A vector *ES*(*g*) can be created by computing *S* for all the annotation terms of *g*. These vectors can be used to quantify the similarity between two genes *g* and $g^{\prime }$ as follows

$$\begin{aligned} \Gamma (g,g^{\prime }) = \frac{ES(g). ES(g^{\prime })}{ ||ES(g)||. ||ES(g^{\prime })|| } \end{aligned}$$

Large value of $\Gamma$ indicate high functional similarity between the two genes *g* and $g^{\prime }$. We compute maximum annotation similarity (MAS), which represents the largest $\Gamma$ value among all possible pairs between a candidate protein *p* and a set of CC genes. A threshold value of 0.75 was used to select candidate CCa proteins.

### Functional annotation of CCa proteins

Each protein identified to be associated with the core circadian clock of *O. tenuiflorum* was then queried against NCBI non-redundant (nr), UniProt^50^ and TAIR^51^ databases using Blast-P^52^ program to assign putative functions. Default parameters except *E* value, which is set to be $10^{-5}$, were used to assign homology. AgriGo^53^ and WEGO^54^ were used to predict Gene Ontology (GO) terms for each CCa protein. KEGG ontology (KO) terms were predicted for each CCa protein using KEGG^55^ database. All the 13 programs aggregated in InterProScan v5.48-83 were utilised to predict protein family, functional domains and sites in each CCa protein^56^.

## Results and discussion

**Figure 1:**
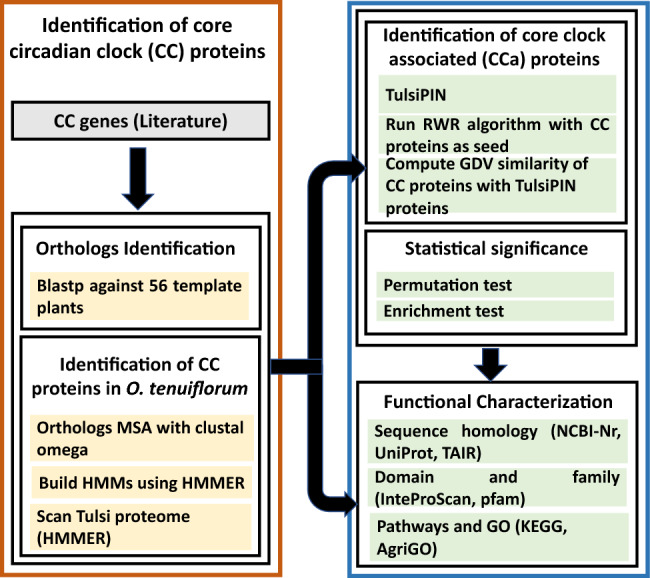
Overall methodology for genome wide identification, characterization of core circadian clock (CC) and core clock associated (CCa) proteins in *O. tenuiflorum*.

*O. tenuiflorum* plants have been reported to produce secondary metabolites (essential oils) which are mixtures of diverse aromatic compounds. These chemical compounds possess enormous medicinal properties that have been exploited continuously since ancient times^31^. Plants are constantly exposed to biotic and abiotic stress under varying environmental conditions, which impede plant growth and development and substantially impact oil production^57,58^. Circadian clock is the main regulatory mechanism that controls the growth and development of any plant^8^. A precisely tuned circadian clock plays a substantial role in increasing the fitness and survival of a plant by temporally coordinating various vital processes, like, metabolism, physiology *etc.* as per the external conditions^8,10^ Thus, we attempt to identify the protein set comprising the core circadian clock and its associated proteins in *O. tenuiflorum* through an exhaustive network science based computational framework presented in Fig. 1.

### Mining and functional characterization of core circadian clock (CC) proteins

We enlisted 24 core circadian clock proteins and attempted to identify them in *Ocimum tenuiflorum*. Proteomes of 56 template plants were scanned using Blast-P for putative orthologs of selected clock proteins that resulted in 2, 543 top hits at an *E* value $< 10^{-5}$. Further, top hits with sequence identity $< 40\%$ and query coverage $< 50\%$ were filtered out, resulting in 963 hits. Separate HMM profiles for each of the CC protein were built by extracting orthologous sequences of that protein from the respective template plants, which satisfy the above criteria. These HMMs were used to scan *O. tenuiflorum* proteome, and based of top hits, all the 24 candidate CC proteins were identified (Table S1). Transcription factors CIRCADIAN CLOCK ASSOCIATED 1 (CCA1) and LONG ELONGATED HYPOCOTYL (LHY) are partially redundant^59^. Also, the transcription regulator LIGHT-REGULATED WD1 (LWD1) is partially redundant with LWD2, LUX ARRHYTHMO (LUX) with NOX, REVEILLE 6 (RVE6) with RVE8^13,60^, so the best hits returned the same proteins for them. These genes are important because they express during different times of the day or form complexes to regulate the expression of other genes. Their functions are synergistic^60,61^, so we selected the second best hit for LWD2, RVE4, PRR5, RVE6, NOX, PSEUDO-RESPONSE REGULATOR 3 (PRR3) and LHY proteins. Functions of all the 24 CC proteins were predicted by querying them against NCBI non-redundant, UniProt and TAIR databases using Blast-P. The results confirmed the identities of predicted clock proteins and reveled that most proteins possess their respective functions (Table S2). Furthermore, protein family and domain databases integrated by InterProScan also confirmed our results (Table S2).

**Figure 2:**
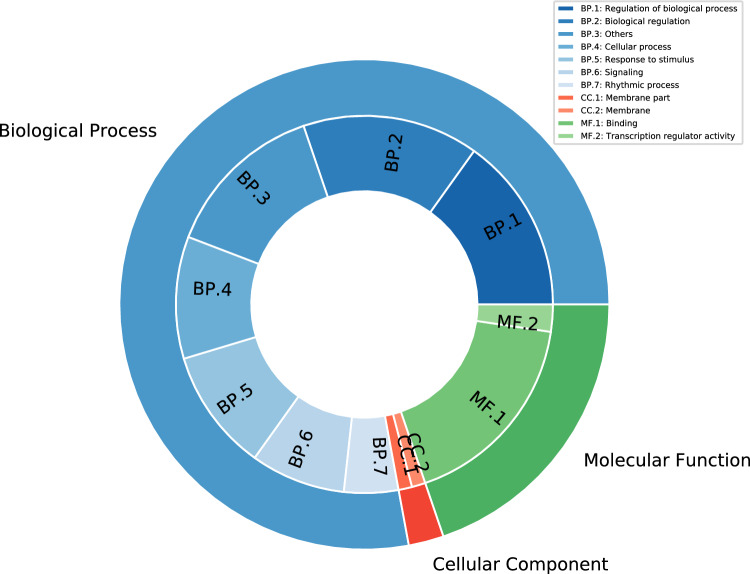
Gene ontology annotations of core circadian clock (CC) proteins of *O. tenuiflorum*.

Functional annotation of CC proteins is followed by their gene ontology (GO) annotations (Table 1). A total of 86 GO terms were successfully identified for these CC proteins, with 67 terms classified into 13 categories of biological processes, 2 terms classified into 2 categories of cellular components, and 17 GO terms associated with 2 categories of molecular functions. ‘Regulation of biological process’, ‘Cellular process’ and ‘Response to stimulus’ are highly enriched among biological processes, followed by ‘signalling’ and ‘rhythmic process’. Among molecular functions, ‘binding’ and ‘transcription regulator activity’ were the most enriched categories (Fig. 2). Successful assignment of “rhythmic process” associated gene ontology (GO) terms to the identified CC proteins further confirms the role of these proteins as the core circadian clock of *O. tenuiflorum*. Furthermore, for finding the CC proteins regulated pathways, the KEGG database was used that predicted KO ids of 16 proteins enriching 8 pathways, of these ‘Circadian rhythms in plants’ is found to be most enriched (Table S2). Other pathways include the ‘Ubiquitin system’ followed by ‘Plant hormone signal transduction’ and ‘Transcription factors’.

**Table 1:** Gene ontology terms and the number of genes associated with them.

**GO term**	**Category**	**Number of proteins**	**Description**
GO:0044425	CC	1	Membrane part
GO:0016020	CC	1	Membrane
GO:0005488	MF	15	Binding
GO:0140110	MF	2	Transcription regulator activity
GO:0051179	BP	1	Localization
GO:0023052	BP	7	Signaling
GO:0009987	BP	9	Cellular process
GO:0050789	BP	13	Regulation of biological process
GO:0050896	BP	9	Response to stimulus
GO:0065007	BP	13	Biological regulation
GO:0008152	BP	2	Metabolic process
GO:0048511	BP	4	Rhythmic process
GO:0048518	BP	1	Positive regulation of biological process
GO:0032501	BP	2	Multicellular organismal process
GO:0032502	BP	2	Developmental process
GO:0022414	BP	2	Reproductive process
GO:0000003	BP	2	Reproduction

### Prediction and functional characterization of core circadian clock (CCa) associated proteins

**Figure 3:**
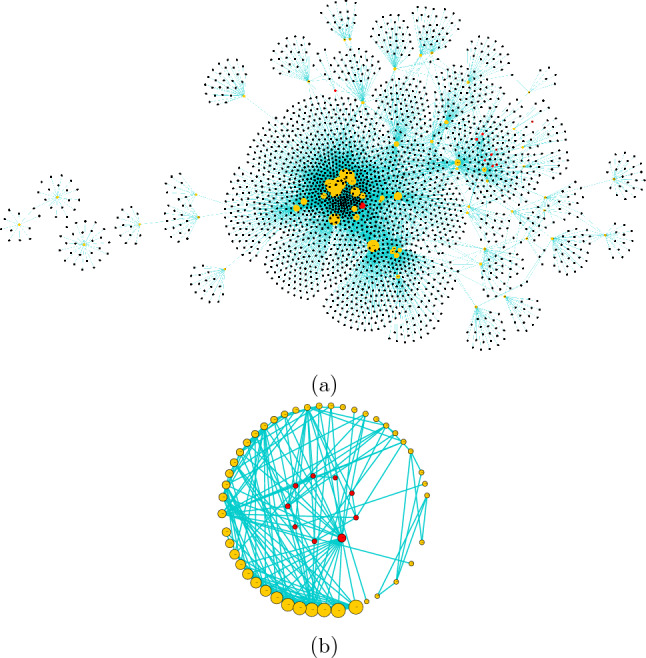
Sub network of proteins obtained by combining core circadian clock (CC) and core circadian associated (CCa) proteins (including first interactors). Yellow colored nodes represent CCa proteins, red colored nodes are CC proteins.

It is known that different centrality measures address different topological aspects of a network and therefore capture the snapshot of a network in terms of selected architectural metric^62^. For example, connectivity-based measures assign higher weights to high degree nodes, whereas middle nodes connecting communities will be ranked higher by measures employing betweenness centrality. Similarly, many graph theoretic methods assign functions to unannotated proteins based on their closeness or shared neighbourhood in the PINs^20,21,63^. However, recently it has been shown that functional similarities among proteins in a PIN are independent of their network location i.e. sharing the same local neighbourhood rather it depends on similarities of the wiring patterns around them^22^. So, in this work, two local network topology based algorithms, namely, random walk with restart (RWR) and graphlet degree vector (GDV), have been leveraged to identify CCa proteins. The GDV based approach does not consider only the current node rather, considers the wiring pattern around that node i.e. it incorporates the information about local neighbourhood^45^. The CC proteins were mapped to the genome-wide interologous protein interaction network of Tulsi (TulsiPIN) to identify circadian clock-associated proteins^37^. A total of 10 proteins were successfully mapped to TulsiPIN proteins which were found to interact with 248 other proteins and form 317 interactions. Since one protein has a degree lower than 3, so removed from the study. The remaining nine CC proteins were used as seed nodes to the RWR algorithm applied on TulsiPIN. This produced a probability value corresponding to each protein representing its likelihood of being a novel circadian clock associated protein. All the proteins having probability values larger than $10^{-5}$ were considered for further study resulting in 733 nodes called $\text {raw}_{RWR}$ proteins (Table S3). Because all RWR proteins can not be circadian clock related, some have been selected due to network architecture alone. We employed permutation test to filter them out and computed a *p* value called the permutation FDR for every RWR protein. All the nodes having *p* values less than 0.05 were selected, resulting in 251 candidate CCa proteins called $\text {candidate}_{RWR}$ proteins and are presented in Table S3. Similarly, we computed pairwise GDV similarity between CC proteins and other TulsiPIN proteins. This resulted in 146 proteins called $\text {raw}_{GDV}$ proteins, having $S_{GDV} > 94$, predicted to be CC associated by the GDV algorithm. Pairwise similarities were obtained from an ensemble of 1000 random networks preserving the degree sequence of TulsiPIN. A total of 146 proteins were found to be statistically significant, having *p* value $< 0.05$ and were called $\text {candidate}_{GDV}$ proteins (Table S3). Furthermore, to add another layer of confidence and select only those proteins with similar functions to CC proteins, we leveraged Gene Ontology and pathways information to compute the functional similarity ($\Gamma$) between each CC protein and every candidate protein. Among 251 $\text {candidate}_{RWR}$ proteins, a total of 43 were found to have $\Gamma > 0.75$ in both GO and pathways information based methods. On the other hand, 25 out of 146 $\text {candidate}_{GDV}$ proteins were found to have $\Gamma$ score 0.75 or above in both methods (Table S3). We combined the proteins obtained from the two methods (RWR and GDV) and found 66 unique candidate proteins, among which 2 were common to both methods. We constructed the circadian connectome of Tulsi by mapping the CC proteins and CCa proteins to TulsiPIN and found that these 90 proteins regulate 2412 other proteins and participate in a total of 8017 interactions (Fig. 3a,b).

**Figure 4:**
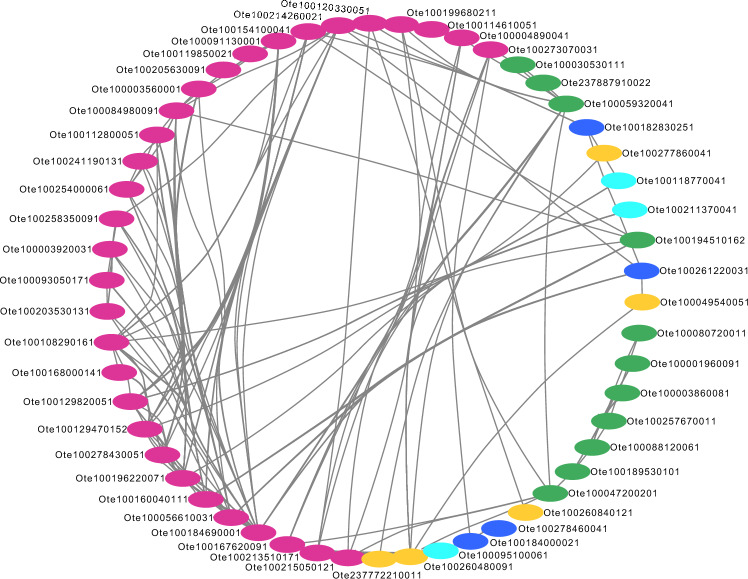
Sub network of circadian clock circuitry including both core circadian clock (CC) and core circadian associated (CCa) proteins only (excluding first interactors). Nodes are colored according to the broad functional class of each protein where hot pink represents proteins involved in gene expression, green (signaling), blue (metabolism), yellow (post translational modification) and cyan represents transport proteins.

Like to CC proteins, all the 66 CCa proteins were queried against NCBI non-redundant, UniProt and TAIR databases using the Blast-P program. The functional annotation of CCa proteins revealed that most proteins are involved in various aspects of growth, development and defence of *O. tenuiflorum* (Table S4). We broadly classified them into five categories, namely, Gene expression (23), metabolism (14), cell signalling (14), transport (9), post translational modification (5) and protein folding (1). It has been shown that approximately one-third of genes in *Arabidopsis thaliana* and $\approx 89 \%$ of its transcripts are regulated by varying environmental conditions, like, temperature, light *etc.*^64,65^. These studies revealed a tight circadian regulation of *A. thaliana* genes at transcriptional level^64^, which is consistent with our results that 20 of CC associated proteins are involved in gene expression and regulation (Fig. 4).

### Circadian regulatory pathway of *O. tenuiflorum*

The basic circadian regulation model comprises a core oscillator that is a complex web of multiple coupled transcriptional and translational feedback loops (TTFL). The core oscillator perceives external cues through input pathways and temporally organises various physiological processes of the plant^66^.

#### The input module of core clock

Proteins Ote100059320041, Ote100047200201 and Ote10021351017 have been predicted to be light perceiving photoreceptors, namely Phytochrome A, Phytochrome B, Adagio protein 1 (ADO1) or ZEITLUPE (ZTL) protein. It is known that phytochromes primarily perceive red and far red light, while cryptochromes and ZEITLUPE (ZTL) family members are blue light photoreceptors. Among the five phytochromes (PHYA-E) in *A. thaliana*, when subjected to continuous red light, PHYA and PHYB are primary contributors to photo perception and entrainment of circadian rhythms^67^. Further, these signals are transduced to the core oscillator through an intermediate protein CONSTITUTIVE PHOTOMORPHOGENIC 1 (COP1)^68^. Proteins Ote237772210011 and Ote100260480091 have been characterised as an E3 ubiquitin ligase COP1. It triggers light gated proteasomal degradation of core clock components ELF3 and GI, directly linking the clock and light perceiving photoreceptors^68,69^. The expression of ELONGATED HYPOCOTYL5 (HY5), FAR RED ELONGATED HYPOCOTYL3 (FHY3) and FAR RED IMPAIRED RESPONSE1 (FAR1) are under tight regulation of PHYA signalling. During the day they bind directly to the ELF4 promoter and induce its expression^70^. The ADO1 and the other two members of this family possess an F-Box domain, a PAS-like LOV domain and six kelch repeats. F-Box domain links target protein for proteasomal degradation by an E3 Ubiquitin ligase complex called skp-cullin-F-Box that is comprised of interactions between F-Box protein with SKP1, CULLIN, and RBX1^71^. It has been shown that ADO1 receive the input signal by interacting with carboxy terminus of blue light photoreceptors phyB and cry1^72^ and mediates blue light dependent degradation of TOC1 and PRR5 through GI mediated stabilization^73^. Ote100194510162 has been characterized as a serine/threonine phosphatase-5 protein. It has been shown to dephosphorylate a specific serine residue on pfr phytochromes. This dephosphorylation enhances phytochrome stability and increases its ability to bind downstream signal transducers and thus enhancing phytochrome mediated photoresponses^74^. Proteins Ote237887910022 and Ote100030530111 have been characterized as serine/threonine phosphatase-7 that has been shown to implicate in cryptochromes mediated signalling^75^. Arabidopsis’s serine/threonine phosphatase-7 (AtPP7) has been shown to act as a nuclear localization signalling molecule that dephosphorylates nuclear signalling intermediates (probably unknown) when exposed to blue light. It acts downstream of the cryptochrome and responds to blue light, ensuring stable signalling^75^.

#### The core circadian clock

**Figure 5:**
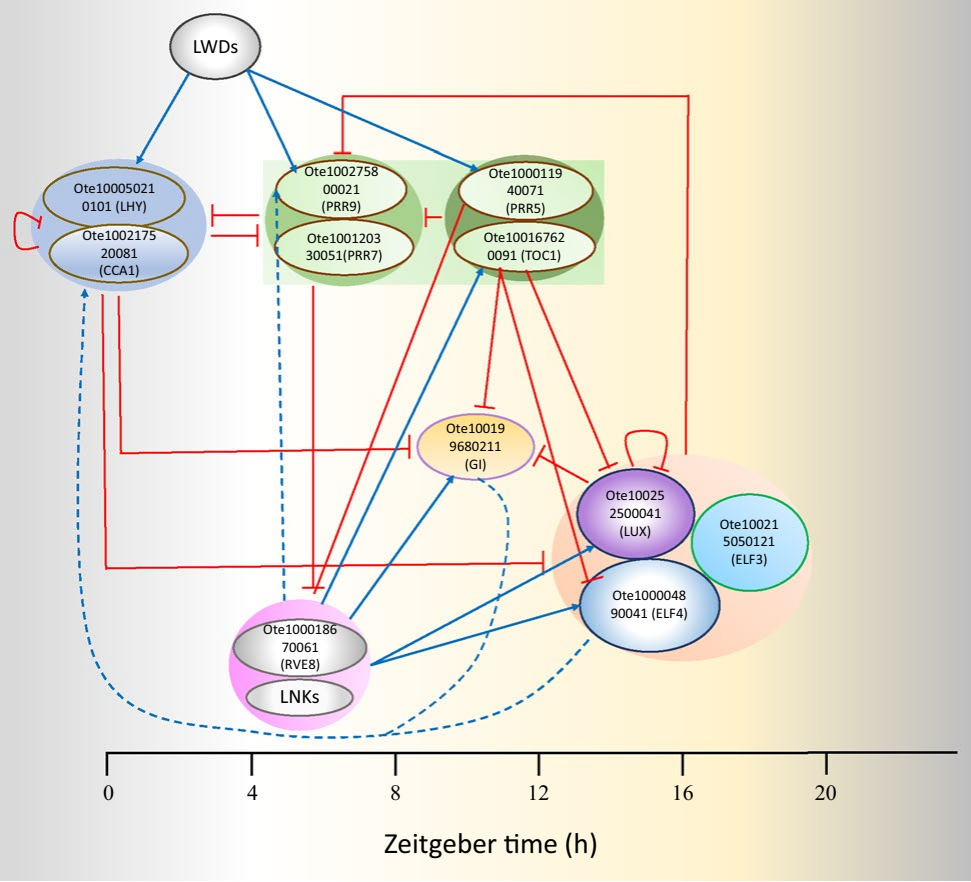
The core clock module of *O. tenuiflorum*.

Proteins CCA1 (Ote100217520081) and LHY (Ote100050210101) represent MYB-related transcription factors that heterodimerize at dawn and repress the expression of morning phased PRR family genes, including PRR9 (Ote100275800021), PRR7 (Ote100120330051) and PRR5 (Ote100011940071). In addition, they repress expression of evening phased genes, including TOC1 (Ote100167620091) and the evening complex (EC) members LUX (Ote100252500041), ELF3 (Ote100215050121), ELF4 (Ote100004890041)^76,77^. The PRR family members are two-component response regulators similar to the ARR family His-Asp phosphorelay systems, except that the aspartate site that accepts the phosphate group is missing. Circadian control at the transcriptional level of all the members of the PRR family has been documented, and they have shown to express sequentially in order of PRR9, PRR7, PRR5, PRR3, and PRR1 within a period of 24*h*^78^. At dawn, CCA1 and LHY negatively regulate PRR1, CCA1 HIKING EXPEDITION (CHE) (Ote237864970011) and their own expression. But during the day, CHE levels rise to repress CCA1, and finally, in the evening TOC1 reset the clock cycle by repressing the expression of CHE^77^. Similarly, PRR7 has also been shown to repress CCA1 and LHY expressions and regulate the expression of other clock components^79^. It acts as the master regulator of various physiological processes of plants, including response to abiotic stress^80^, photoperiodic flowering and temperature compensation^79^. TOC1 (PRR1) also repress the expression of PRR5, LUX, EARLY FLOWERING 4 (ELF4) and GIGANTEA (GI) (Ote100199680211)^79^. EC component express from evening onwards throughout night and are required for rhythmic expression of CCA1 and LHY. LUX downregulates the expression of PRR9 and itself by binding the LUX binding sites (LBS) in their promoters. EC is believed to indirectly upregulate the expression of CCA1 and LHY by repressing the expression of PRR9, PRR7 and TOC1^81^. REVEILLE family members, including RVE8 (Ote100018670061), RVE4 (Ote100114610051) along with coactivators NIGHT LIGHT-INDUCIBLE AND CLOCK-REGULATED 1 (LNK1) (Ote100153880201) and LNK2 (Ote100241100111) induce the expression of PRR5, TOC1, LUX and ELF4^82,83^. Figure 5 presents the core clock identified in Tulsi.

#### The output pathways regulated by core clock

Ote100001960091 and Ote100189530101 have been identified as Ethylene receptor-1 and ethylene response sensor-1, respectively. Both encode histidine kinases similar to members of the two-component regulator family and have been shown to be integral components in ethylene signalling^84^. The gaseous hormone has many implications, like, fruit-ripening, senescence, stress response, seed germination and many more, and its signalling has been shown to be under circadian control^85^. Protein Ote100058660011 has been predicted as Gibberellin (GA) receptor GID1B that acts as a soluble gibberellin receptor. GA is a diterpenoid hormone that regulates various stages of plant growth and development, like, seed germination, development of fruit, pollen tube and flowering time. GID1 binds with biologically active GAs like GA1, GA3, and GA4 and targets a GRAS family protein DELLA that negatively regulates GA signalling for proteasomal degradation^86^. Proteins Ote100237240051 and Ote100260840121 are predicted to be UDP-N-acetylglucosamine–peptide N-acetylglucosaminyltransferase SPINDLY (SPY) that negatively regulate Gibberellin signalling to inhibit hypocotyl elongation. It has been shown that SPY and GI function together, and GI acts as a light gated negative regulator of SPY function to control various physiological processes like elongation of hypocotyl, flowering time and cotyledon movements^87^. Ote100003860081, predicted as a two-component response regulator ARR3 that behaves like a bacterial two-component system that negatively regulates cytokine signalling and affects the phosphorylation state of ARRs by transducing the signal through a multi-step His-Asp phosphotransfer process^88^. Ote100257670011 has been characterized as a Histidine kinase-3 protein that acts as a positive regulator of cytokine signalling^89^. Reactive oxygen species (ROS) production, signalling and scavenging have been shown to be linked with circadian clock output and plant response to oxidative stress^90^. Ote100224900091 has been characterized as an ascorbate oxidase that oxidizes ascorbate to MDA and reduces $\text {O}_{2}$ to $\text {H}_2\text {O}$^91^. Protein Ote100214260021 has been characterized as a regulator of nonsense transcripts UPF2 that participates in nonsense mediated decay (NMD) pathway and regulates the degradation of mRNA containing premature termination codons^92^. It has been shown to be involved in photoperiod dependent transcriptional regulation of jasmonic acid and salicylic acid induced defence genes LOX2 and VSP2 in response to biotic and abiotic stresses^92^.

## Summary and conclusions

Considering the importance of circadian rhythms in regulating the physiological processes of the plant, the current study reports a novel network based methodology that exploits the topological information of a network to identify circadian connectome, including the core clock (CC) proteins and clock associated proteins. Although we have implemented the proposed framework in *O. tenuiflorum*, but we can easily adapt it to any other organism having an endogenous clock. It will increase our understanding of various components of circadian circuitry responsible for the circadian rhythms and the underlying mechanisms sustaining these rhythms, which are largely unknown in Tulsi. This study proposes a methodology to identify 24 known core circadian clock genes in Tulsi that leverage in house built HMMs from 56 template plants. Further, we propose a hybrid methodology that combines RWR and GDV local topological measures to identify novel proteins associated with the core clock. Based on RWR, GDV, and two statistical significance tests 70 putative core clock associated genes proteins were obtained. Functional annotation of the candidate proteins was performed, and we found that many proteins are involved in the regulation of gene expression. We obtained crucial proteins transducing red, far-red and blue light signals to the core oscillator. Furthermore, we obtained proteins involved in different physiological processes, like, gibberellin signalling, ethylene signalling, cytokine signalling, abiotic and biotic stress response etc. We hope that the proposed framework for reporting genome-wide identification and characterization of the core circadian clock and associated proteins in *O. tenuiflorum* shall pave the way for prioritizing the CC or any other biological process associated proteins in plants.

### Supplementary Information

Supplementary Information.

## Data Availability

All data is available in the manuscript or the supplementary materials and codes used are available at GitHub https://github.com/vikramsinghlab/Pcrp.
